# Combining Low-dimensional Wavelet Features and Support Vector Machine for Arrhythmia Beat Classification

**DOI:** 10.1038/s41598-017-06596-z

**Published:** 2017-07-20

**Authors:** Qin Qin, Jianqing Li, Li Zhang, Yinggao Yue, Chengyu Liu

**Affiliations:** 1 School of Instrument Science and Engineering, Southeast University, Nanjing, 210018 P.R. China; 2 Computational Intelligence Research Group, Department of Computing Science and Digital Technologies, Faculty of Engineering and Environment, University of Northumbria, Newcastle, NE1 8ST UK

## Abstract

Automatic feature extraction and classification are two main tasks in abnormal ECG beat recognition. Feature extraction is an important prerequisite prior to classification since it provides the classifier with input features, and the performance of classifier depends significantly on the quality of these features. This study develops an effective method to extract low-dimensional ECG beat feature vectors. It employs wavelet multi-resolution analysis to extract time-frequency domain features and then applies principle component analysis to reduce the dimension of the feature vector. In classification, 12-element feature vectors characterizing six types of beats are used as inputs for one-versus-one support vector machine, which is conducted in form of 10-fold cross validation with beat-based and record-based training schemes. Tested upon a total of 107049 beats from MIT-BIH arrhythmia database, our method has achieved average sensitivity, specificity and accuracy of 99.09%, 99.82% and 99.70%, respectively, using the beat-based training scheme, and 44.40%, 88.88% and 81.47%, respectively, using the record-based training scheme.

## Introduction

Electrocardiogram (ECG) provides detailed information on a patient’s heart status. Cardiac arrhythmias are groups of conditions in which the electrical activity of the heart is irregular, faster or slower than normal, or even waveform malformation^1^. Any disorder of heart rhythm or alteration in morphological pattern is an indication of some underlying pathology, which could be detected by analyzing the ECG waveforms. However, clinical analysis and diagnosis using ECG signals by physicians, especially for long-term monitoring cases, are extremely time-consuming and sometimes even unrealistic or inaccessible to remote areas. Consequently, automatic arrhythmia beat classification is urgently required although challenging for dynamic ECG processing.

There are three main steps in arrhythmia beat classification, namely, feature extraction, feature selection, and classifier construction^2–4^. As a premise of classification, feature extraction from ECG signals is an important and preliminary step since reliable and robust classification relies on effective feature representations. An ECG feature can be defined as a distinctive or characteristic measurement, extracted from a beat episode used to discriminate its type^5, 6^. Features are expected to represent patterns with purpose of minimizing the loss of essential information. Generally, they are performed either in time domain to obtain morphological features^7–12^, or in frequency domain to discover changes in power spectrum of ECG waves^13–19^. Or they are used in time-frequency domain to exhibit simultaneously morphological and spectral features^20–24^.

In the related research, numerous feature extraction techniques have been developed. In^2^, a combination of linear and nonlinear features was used as input to the support vector machine (SVM) classifier with a radial basis function (RBF) kernel. Using a 10-fold cross validation method, sensitivity of 98.91%, specificity of 97.85% and accuracy of 98.91% were reported for the classification of five types of arrhythmia when evaluated using a set of 110094 beats from MIT-BIH arrhythmia database (MITDB). In^5^, four features (AC power, kurtosis, skewness and RR interval ratio) were extracted from each QRS complex by using the fourth and fifth decomposition level derived from dual tree complex wavelet transform. In this technique, the multi-layer back propagation neural network (BPNN) was trained by using the first three-minute signals of each ECG record, and the remaining 27-minute signals of each ECG record were used for test. An overall sensitivity of 94.64% was achieved. The work of^23^ employed principal component analysis (PCA) to extract discrete cosine transform (DCT) coefficients from segmented ECG beats as input features for propagation neural network (PNN)-based classification. Their method has obtained an average accuracy rate of 99.52%, sensitivity of 98.69%, and specificity of 99.91% using 10-fold cross validation for the classification of six types of mixed heartbeats. The work of^25^ adopted morphological and temporal features to classify six types of ECG beats, i.e. normal (N), atrial premature contraction (A), premature ventricular contraction (V), right bundle branch block (R), left bundle branch block (L) and paced (P) beats. The work employed particle swarm optimization (PSO)-based feature optimization and SVM-based classification. Tested with a set of randomly selected beats over three trials, an overall accuracy of 89.72% was achieved for the evaluation of 40438 test beats from 20 MITDB ECG records. The work of^26^ took advantage of a similar method as the one illustrated in^25^ to classify another six types of beats. Nevertheless, their experimental results were limited owing to the usage of a small dataset from MITDB for evaluation. It was also unclear whether the training and test sets are contributed by the same individuals, or otherwise. Based on the statistical theory, the work of^27^ described a novel feature extraction method using higher order statistics of wavelet packet coefficients. Five beat classes from MITDB were recognized by the *k*-nearest neighbors (*k*-NN) algorithm. Their method employed independent 3345 and 2542 beats for training and test, respectively. It obtained 90% for sensitivity, 92% for selectivity and 98% for specificity. According to^28, 29^, among all the methods, wavelet transform is still considered as the most efficient and prevalent tool for ECG feature extraction. Due to the non-stationary property of ECG signals, the inherent properties of wavelet transform include excellent time-frequency location and cross sub-band similarity for such types of signals^30^.

There are many studies focusing on demonstrating the effective measures for ECG feature extraction. However, the problem of dimensionality reduction has rarely been explored^31^. Generally, a large number of features will benefit the classifier to construct classification model with comprehensive knowledge over the training samples. Meanwhile, a large number of features also increase the computational complexity^32^. A superior feature vector should contain optimal elements that describe the critical characteristics of a signal with less redundancy. Although wavelet features can efficiently provide a comprehensive description in time-frequency domain, some of the coefficients may contain redundant information. Currently, the most classical dimensionality reduction algorithm is PCA^29, 33^. Apart from it, researchers have proposed many other advanced feature selection techniques for dimensionality reduction. In^20^, an adaptive feature selection system for ECG wavelet coefficient was established by sorting feature priority. The system has achieved an improved recognition rate of 98.92% for the evaluation of a set of randomly selected 100441 beats from MITDB using a modified SVM classifier. However, the feature dimension in their work was still high, i.e. greater than 50. Unlike conventional methods, a Teager energy-based ECG feature extraction scheme was presented in^34^. Only two features were exhibited for neural network-based classification. The scheme has realized an average classification accuracy of 95% over the evaluation of 67960 beats. In^35^, the authors compared three dimensionality reduction methods, i.e. PCA, linear discriminant analysis (LDA) and independent component analysis (ICA), on wavelet coefficients. It was shown that ICA integrated with a PNN classifier achieved the best performance, i.e. 99.28%, 97.97%, and 99.83% for the average accuracy rate, sensitivity and specificity, respectively, for the classification of five types of beat classes.

Apart from the aforementioned issues, there is another important problem rarely illustrated in the published literatures. Most of the ECG feature extraction techniques verify the feature performance by the application of classifiers with training and test set randomly selected from the database, or a certain fraction of each class is selected as the training set and the remaining heartbeats are used as the test set. However, it should be noted that this classification scheme is not a realistic performance measure of automatic heartbeat classification in real-world applications. It leads to optimistic results since the inter-individual variation in ECG characteristics is less in such tests because some of the training and test beats may come from the same patient. This weighs against the principle of pattern recognition that the training and test beats should be completely from different individuals, and few researchers have demonstrated the detailed research addressing this issue.

To these ends, a wavelet based feature extraction and PCA based feature reduction method was proposed in this study. Then we used SVM for arrhythmia beat classification with a 10-fold cross validation. Two training schemes, i.e., beat-based and record-based schemes, were used for classification evaluations.

The remainder of the paper is organized as follows. Section 2 elaborated the detailed procedure of the proposed algorithm, along with the dataset and the evaluation indices. Section 3 demonstrated the classification results over two different training schemes. Section 4 compared our method with several recent developments, where feasible measures were also discussed to improve the classification performance on imbalanced beat distribution. Finally, the summarization of this study was presented in Section 5.

## Methods

### Dataset

The MITDB comprises of 48 ECG records and each record contains a 30-minute ECG signal. The signals are sampled at 360 Hz with 11-bit resolution over a 10 mV range and band-pass filtered at 0.1~100 Hz^36^. The ECG records from this database include signals with acceptable quality, sharp and tall P and T waves, negative R waves, small R-peak amplitudes, wider R waves, muscle noise, baseline drift, sudden changes in beat morphology, multiform V beats, long pauses and irregular heart rhythms^37^. In this study, the Lead II ECGs in each record are used. The arrhythmia annotations are provided for each ECG beat (annotated at the R-peak locations) from the database. There are up to 16 different types of arrhythmia. In this study, only six types (A, L, N, P, R and V beat) are used since these beats occupies the majority of the database (107049 out of a total of 109966).

Ten-fold cross validation scheme is used for the evaluation of this research. For arrhythmia classification, the most frequently employed cross validation scheme is randomly and equally selecting the beats from each classification type for each folder, i.e., the beat-based cross validation scheme. However, this training scheme may result in over-fitting problem since the training and test beats can come from the same record. i.e., from the same patient. While the record-based cross validation scheme can avoid the above over-fitting problem since all beats in the test set completely come from unknown patients (i.e. different individuals). Thus, both beat-based and record-based cross validation schemes are adopted in this study. Tables 1 and 2 illustrate the data profiles for the two schemes. Table 3 shows the record division of the training and test sets for the record-based cross validation scheme demonstrated in Table 2. It should be noted that some of the beat types only exist in several records (e.g. 4 records containing the L beat; 6 records containing the R beat, and 4 records containing the V beat). As a result, these records may be used more than once as the test data in cross validation.

**Table 1:** Data profile for the beat-based 10-fold cross validation scheme.

Folder	Number of beats in training set						Total training beats	Number of beats in test set						Total test beats
	A	L	N	P	R	V		A	L	N	P	R	V	
1	2292	7265	67520	6323	6530	6417	96347	254	807	7502	702	725	712	10702
2	2292	7265	67520	6323	6530	6416	96346	254	807	7502	702	725	713	10703
3	2292	7265	67520	6323	6530	6416	96346	254	807	7502	702	725	713	10703
4	2292	7265	67520	6323	6530	6416	96346	254	807	7502	702	725	713	10703
5	2291	7265	67520	6323	6530	6416	96345	255	807	7502	702	725	713	10704
6	2291	7265	67520	6322	6529	6416	96343	255	807	7502	703	726	713	10706
7	2291	7265	67520	6322	6529	6416	96343	255	807	7502	703	726	713	10706
8	2291	7265	67520	6322	6529	6416	96343	255	807	7502	703	726	713	10706
9	2291	7264	67519	6322	6529	6416	96341	255	808	7503	703	726	713	10708
10	2291	7264	67519	6322	6529	6416	96341	255	808	7503	703	726	713	10708

**Table 2:** Data profile for the record-based 10-fold cross validation scheme.

Folder	Number of beats in training set						Total training beats	Number of beats in test set						Total test beats
	A	L	N	P	R	V		A	L	N	P	R	V	
1	2414	5581	70682	5483	5090	6912	96162	132	2491	4340	1542	2165	217	10887
2	2544	5949	70989	4947	5725	6461	96615	2	2123	4033	2078	1530	668	10434
3	2427	6615	68715	5647	7170	6979	97553	119	1457	6307	1378	85	150	9496
4	2544	6071	70167	4998	5430	6760	95970	2	2001	4855	2027	1825	369	11079
5	2545	5581	70719	4947	6002	7027	96821	1	2491	4303	2078	1253	102	10228
6	1128	5949	70189	5483	6858	6503	96110	1418	2123	4833	1542	397	626	10939
7	1960	6615	69882	4998	5005	6809	95269	586	1457	5140	2027	2250	320	11780
8	2534	6071	69583	5647	5725	6596	96156	12	2001	5439	1378	1530	533	10893
9	2231	4124	72718	5483	7170	6824	98550	315	3948	2304	1542	85	305	8499
10	2539	5949	71772	4998	5430	6294	96982	7	2123	3250	2027	1825	835	10067

**Table 3:** Record division of the training and test sets for the record-based cross validation scheme.

Folder	Training set	Test set	Folder	Training set	Test set
1	All other records	100, 101, 109, 118, 217	6	All other records	111, 202, 203, 217, 232
2	All other records	105, 106, 107, 111, 124	7	All other records	102, 118, 207, 209, 210
3	All other records	104, 112, 113, 114, 207	8	All other records	104, 124, 214, 215, 219
4	All other records	102, 116, 117, 212, 214	9	All other records	109, 207, 217, 222
5	All other records	107, 109, 122, 123, 231	10	All other records	102, 111, 212, 233

### Method description

**Figure 1:**
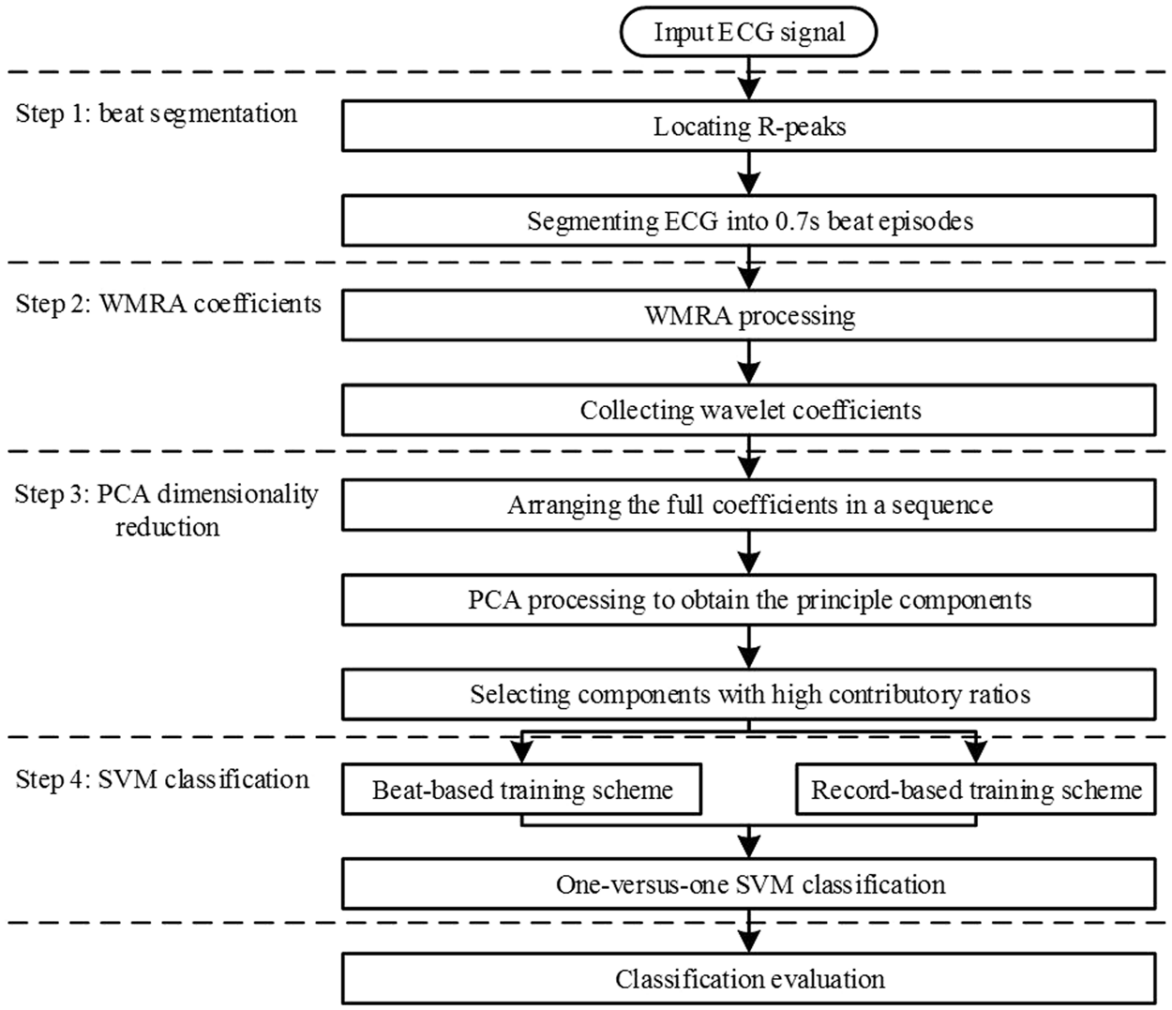
Block diagram of the proposed feature extraction algorithm.

The flowchart of the proposed method is shown in Fig. 1. In Step 1, an ECG signal was segmented into 0.7 s episodes based on the R-peak locations provided by MITDB. In Step 2, each 0.7 s ECG episode was analyzed by wavelet multi-resolution analysis (WMRA), and thus the wavelet features were generated. In Step 3, PCA was used to reduce the feature dimension to generate the low-dimensional wavelet features. In Step 4, both beat-based and record-based cross validation schemes were used for the training of the SVM model, and the corresponding accuracies of arrhythmia beat classification were obtained.

### Step 1: beat segmentation

**Figure 2:**
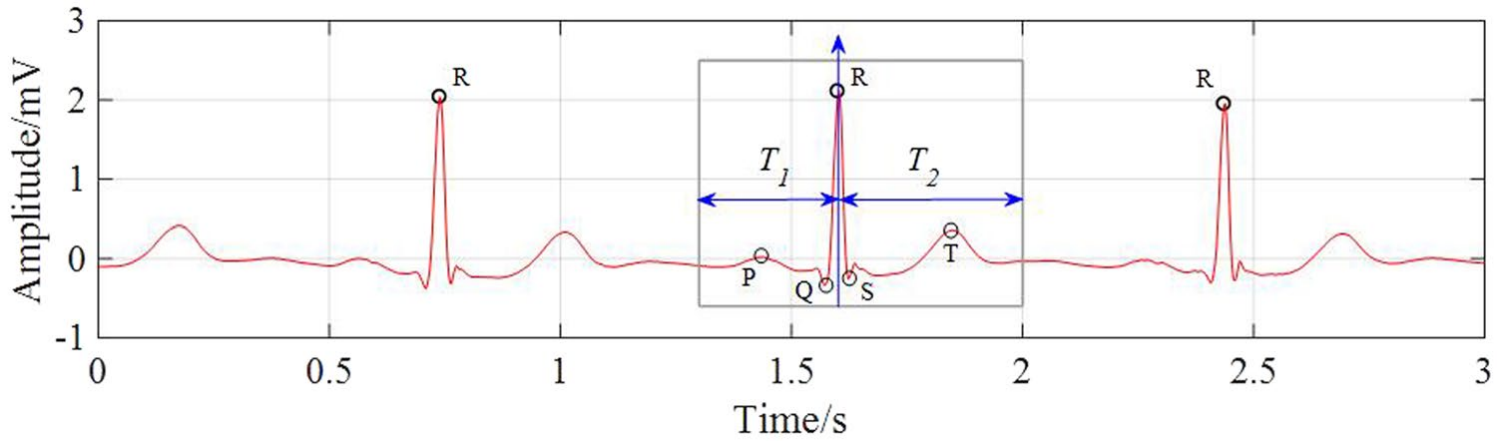
Beat segmentation of a typical ECG signal.

ECG beat segmentation is the process of intercepting multiple points in the signal so that not only successive beats are separated, but also the waveforms embedded in every beat are distinguished^38–40^. This definition clarifies two types of ECG beat features: single-beat and multiple-beat features. The former refers to the features that are extracted from a single beat, which usually contains one and only one R-peak. Meanwhile, the latter refers to the features that are dependent on at least two successive beats. These features include more information than one R-peak. Typical waveforms and beat segmentation on an ECG signal are illustrated in Fig. 2. As can be seen, the positions of P wave, QRS complex and T wave are related directly to the location of the R-peak, which is generally regarded as the segmentation symbol.

According to^41^, the general frequency of ECG rhythm is between 60 and 80 per minute, hence, a 0.25 s offset before R-peak is adopted as the *T*_1_ duration and a 0.45 s offset after R-peak is adopted as the *T*_2_ duration, resulting in a length of 0.7 s (252 points with sampling frequency of 360 Hz as illustrated in^37^) signal segment for each beat to cover the P wave, QRS complex and T wave.

### Step 2: WMRA coefficients

WMRA enhances the signal by extracting variable information with different translation and shrinkage scales. It is very suitable to process an ECG signal that is non-stationary of small amplitude (0.01~5 mV) and low frequency (0.05~100 Hz)^42^. This technique also provides a high reduction in computational time^43^. By using WMRA, frequency bands below 0.05 Hz and above 100 Hz can be excluded. Simultaneously, some interference with frequency concentrated in these bands can be removed^44^. In addition, according to the Nyquist criterion, sub-frequency band presented by each decomposition level is directly related to the sampling rate^43^. Consequently, the ECG signals, sampled at 360 Hz, are decomposed up to 8 levels in this study.

**Figure 3:**
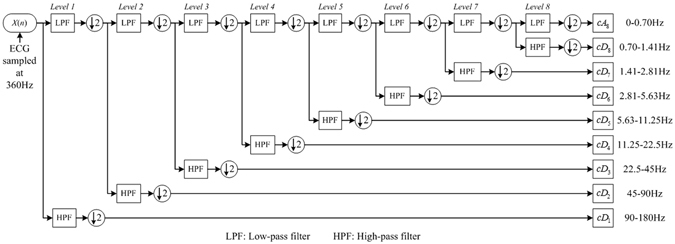
The decomposition process of the 8-level WMRA.

Figure 3 shows the decomposition procedure of 8-level WMRA using *bior*6.8 wavelet (the reason for choosing this wavelet is illustrated in Supplementary Appendix). WMRA decomposes the sampling frequency by a factor of 2 into high frequency band of detail coefficient (*cD*_*j*_) and low frequency band of approximation coefficient (*cA*_*j*_), both in *Level j*. The decomposition is repeated until the continuous sub-frequency bands contain the ECG frequency interval of 0.05~100 Hz. Among the wavelet coefficients, *cD*_1_ ∼ *cD*_8_ consist of frequency components in range of 0.70-180 Hz, which is the ECG frequency band of interest. *cA*_8_ with frequency band 0~0.70 Hz is beyond the ECG frequency. It is not the employed coefficient containing baseline drift and other interference. Consequently, *cA*_8_ is neglected and *cD*_1_ ∼ *cD*_8_ are preserved as original features.

### Step 3: PCA dimensionality reduction

PCA uses an orthogonal transformation to convert a set of observations of possibly correlated variables into a set of values of linearly uncorrelated variables called principal components. It can be done by eigenvalue decomposition of a data covariance matrix or singular value decomposition of a data matrix. This transformation is defined in such a way that the first principal component has the largest possible, and each succeeding component in turn has the highest variance possible under the constraint that it is orthogonal to the preceding components. The results of a PCA are usually discussed in terms of component scores. If a multivariate dataset is visualized as a set of coordinates in a high-dimensional data space, PCA can produce a lower-dimensional feature viewed from its most informative viewpoint^35^.

**Figure 4:**
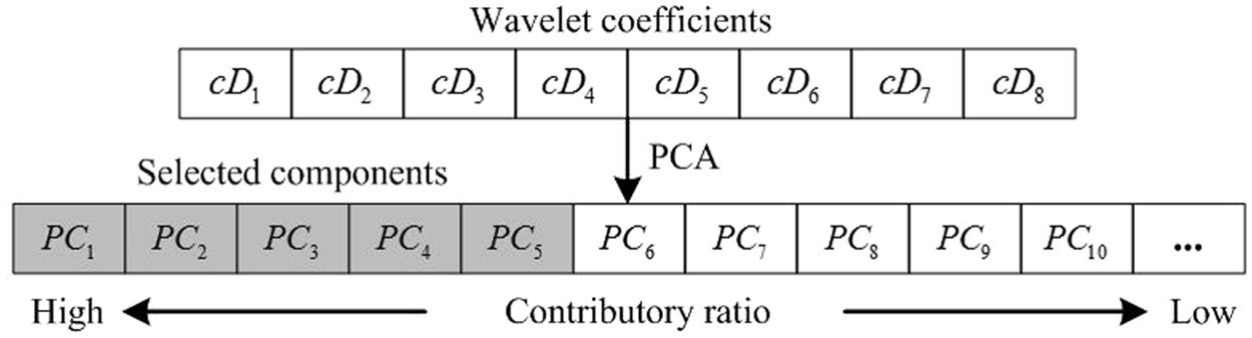
Low-dimensional feature vector generated by PCA using wavelet coefficients.

The total number of wavelet coefficients in all decomposition levels depends on the beat length *L*. As illustrated in Fig. 3, when down sampling, the frequency is divided into two complementary intervals, and the signal is intercepted into half of the original length. After 8-level WMRA, the total number of detail coefficients will be at least $(\frac{1}{{2}^{1}}+\frac{1}{{2}^{2}}+\ldots +\frac{1}{{2}^{8}})L$. Obviously, not all the coefficients are necessary to form the input features owing to the redundancy that may result in high time consumption in classification procedure. The original features are arranged into a temporary vector with *cD*_1_ as the beginning and *cD*_8_ as the ending, as shown in Fig. 4. PCA reduces the dimension by using the first few principal components. For example, if the accumulated contributory ratio of the first five principle components has already reached 95% representing or spanning in the whole feature space, then, the five components are taken as the new features.

### Step 4: SVM classification

One-versus-one SVM, which can be implemented in multi-classification applications, is an enhanced classifier derived from SVM^45^. Since the inherent property of SVM can only determine one hyperplane of two classes. The hyperplane corresponding to each class should be trained individually. By using a popular SVM training tool that determines the support vectors for each hyperplane, the remaining process is to reach the final decision for each class based on the classification results of all hyperplanes.

One-versus-one SVM uses the majority voting scheme to categorize all the hyperplanes, with which the classification result is determined by selecting the maximum likelihood class. For a test set ***S***, if there are *n* classes, the total number of hyperplanes constructed among these classes is *n*(*n*−1)/2. The class having the most votes given by all the hyperplanes is recognized as the outcome of the corresponding hyperplane. For instance, if the classification output of a hyperplane indicates that the input set ***S*** should be in class ***C***, then class ***C*** gets one vote from this hyperplane. The set ***S*** is predicted to be in class ***C*** if this class get the maximum number of votes *φ*(***C***, ***S***), which is defined as

1 $$\phi ({\boldsymbol{C}},\,{\boldsymbol{S}})=\sum _{p={\rm{1}}}^{n(n-1)/2}\nu (p,{\boldsymbol{C}},{\boldsymbol{S}})$$

2 $$\nu (p,{\boldsymbol{C}},{\boldsymbol{S}})=\{\begin{array}{l}1\,{\rm{if}}\,\delta (p,{\boldsymbol{S}})={\boldsymbol{C}}\\ 0\,{\rm{otherwise}}\,\end{array}$$

where *p* denotes the index of a hyperplane, while *v*(p, ***C***, ***S***) represents the Boolean function for the vote corresponding to the *p*-th hyperplane, and *δ*(*p*, ***S***) is the class index of the classification result for the *p*-th hyperplane.

### Evaluation method

The classification performance is evaluated using sensitivity (*SEN*), specificity (*SPE*) and accuracy (*ACC*). The three measures of one beat type ***H*** are expressed in Eqs (3)~(5).

3 $$SEN=\frac{{\sum }_{H={\rm{1}}}^{K}T{P}_{H}}{{\sum }_{H={\rm{1}}}^{K}T{P}_{H}\,+\,{\sum }_{H={\rm{1}}}^{K}F{N}_{H}}$$

4 $$SPE\,=\,\frac{{\sum }_{H={\rm{1}}}^{K}T{N}_{H}}{{\sum }_{H={\rm{1}}}^{K}T{N}_{H}\,+\,{\sum }_{H={\rm{1}}}^{K}F{P}_{H}}$$

5 $$ACC\,=\,\frac{{\sum }_{H={\rm{1}}}^{K}T{P}_{H}\,+\,{\sum }_{H={\rm{1}}}^{K}T{N}_{H}}{{\sum }_{H={\rm{1}}}^{K}T{P}_{H}\,+\,{\sum }_{H=1}^{K}T{N}_{H}\,+\,{\sum }_{H={\rm{1}}}^{K}F{P}_{H}\,+\,{\sum }_{H={\rm{1}}}^{K}F{N}_{H}}$$

where *K* is the number of beat types; *TP*_***H***_ (true positives) is the number of ***H*** types that are correctly classified; TN_***H***_ (true negative) is the number of not-***H*** types that are correctly classified; *FP*_***H***_ (false positive) is the number of not-***H*** types that are incorrectly predicted as ***H*** types; and *FN*_***H***_ (false negative) is the number of ***H*** types that are incorrectly predicted as not-***H*** types. For instance, the four indices of N beat, i.e. *TP*_*N*_, *TN*_*N*_, *FP*_*N*_ and *FN*_*N*_, are defined in Table 4. *TP*, *TN*, *FP* and *FN* of other beats can be defined in a similar way.

**Table 4:** Instructions for the definitions of *TP*, *TN*, *FP*, and *FN* for N beat.

	A	L	N	P	R	V
A	*TN*_*N*_	*TN*_*N*_	*FP*_*N*_	*TN*_*N*_	*TN*_*N*_	*TN*_*N*_
L	*TN*_*N*_	*TN*_*N*_	*FP*_*N*_	*TN*_*N*_	*TN*_*N*_	*TN*_*N*_
N	*FN*_*N*_	*FN*_*N*_	*TP*_*N*_	*FN*_*N*_	*FN*_*N*_	*FN*_*N*_
P	*TN*_*N*_	*TN*_*N*_	*FP*_*N*_	*TN*_*N*_	*TN*_*N*_	*TN*_*N*_
R	*TN*_*N*_	*TN*_*N*_	*FP*_*N*_	*TN*_*N*_	*TN*_*N*_	*TN*_*N*_
V	*TN*_*N*_	*TN*_*N*_	*FP*_*N*_	*TN*_*N*_	*TN*_*N*_	*TN*_*N*_

### Data availability

The datasets generated and analyzed during the current study are available from the corresponding author on reasonable request. The raw ECG datasets used during the study are available in the MIT repository (http://physionet.org/cgi-bin/atm/ATM).

## Results

### Results of feature selection using PCA

**Figure 5:**
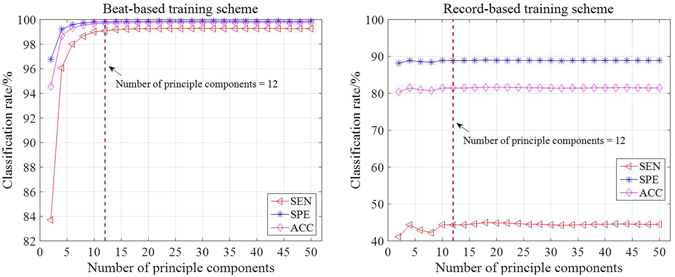
Classification rates with different numbers of principle components.

After applying WMRA, a total of 373 candidate features were generated for dimensionality reduction by PCA. Figure 5 shows the average results of *SEN*, *SPE* and *ACC* using the 10-fold cross validation for the two schemes. The tested numbers of principle components are evens from 2 to 50. For the beat-based scheme, with the increase of the number of principle components, the classification accuracy firstly increases rapidly and then remains at stable levels using 12 or more principle components. For the record-based scheme, classification accuracy also remains at stable levels using 12 or more principle components. Thus, we used 12-element input features for the subsequent beat-based and record-based cross validation schemes.

### Results of beat-based 10-folder cross validation scheme

**Figure 6:**
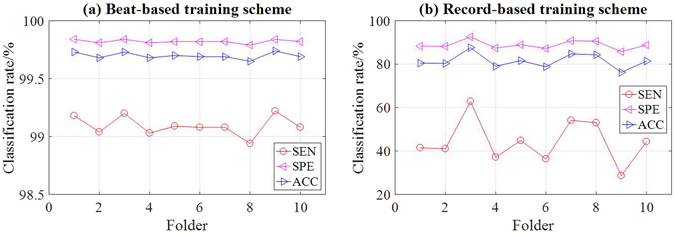
Classification performance for the beat-based (**a**) and record-based (**b**) schemes using 10-fold cross validation.

One-versus-one SVM is implemented using RBF with the Gaussian kernel. We used the recommended values for the parameter settings, i.e. *C* = 10 and *γ* = 0.1, as suggested in^45^. Figure 6a displays the classification results of the beat-based 10-fold cross validation, and the total results are summarized in Table 5, which shows that the classification with the 12-element feature vectors has achieved 99.09%, 99.82% and 99.70% for *SEN*, *SPE* and *ACC*, respectively (the detailed results are shown in Supplementary Tables S1 and S2). Specifically, P beats achieve the highest classification accuracy among all types. All P beats in the 3rd, 4th, 9th folders are correctly recognized. In addition, the classification accuracies for V beats exceed 99.50% for each folder. However, the classification sensitivities for A beats are lowest since the waveforms of A beats are extremely similar as those of N beats.

**Table 5:** Classification Results for the 10-fold cross validation.

	Beat type	Beat-based training scheme			Record-based training scheme		
		*SEN* (%)	*SPE* (%)	*ACC* (%)	*SEN* (%)	*SPE* (%)	*ACC* (%)
Mean	A	83.35	99.91	99.52	0.76	93.98	91.63
	L	99.32	99.97	99.92	0.00	99.44	77.68
	N	99.67	98.14	99.21	90.79	53.19	70.18
	P	99.87	100.0	99.99	11.00	99.95	85.02
	R	99.27	99.96	99.92	4.17	99.96	88.46
	V	97.45	99.79	99.63	74.32	75.83	75.82
	**Total**	**99.09**	**99.82**	**99.70**	**44.40**	**88.88**	**81.47**
Standard deviation	A	1.76	0.03	0.05	1.26	5.78	5.42
	L	0.21	0.02	0.02	0.00	1.07	8.77
	N	0.07	0.21	0.07	10.82	18.45	10.11
	P	0.10	0.01	0.01	17.78	0.08	3.43
	R	0.31	0.02	0.03	12.42	0.03	7.18
	V	0.67	0.04	0.07	10.59	15.12	14.58
	**Total**	**0.08**	**0.02**	**0.03**	**9.46**	**1.89**	**3.15**

### Results of record-based 10-folder cross validation scheme

The recognition rates of record-based scheme are much worse compared to those of the beat-based scheme, as demonstrated in Fig. 6b. The classification rates are no more than 90% for all beat types. The average *SEN*, *SPE* and *ACC* are 44.40%, 88.88% and 81.47% respectively (the detailed results are shown in Supplementary Tables S3 and S4) as illustrated in Table 5. The most significant characteristics in Table 5 are the recognition results of N beat, which has the highest *SEN* but lowest *SPE* and *ACC* among the six types of beats. Namely, the N beat classification has large *TP* values but small *TN* values. The phenomenon indicates that the majority of beats are recognized as N beats due to the imbalanced data. In MITDB, each beat type excluding the N beat is greatly outnumbered by the N beat, which occupies a majority class of approximately 70%. As a result, SVM tends to sort through huge populations of beats to find the small number of beats.

## Discussions

By exploring the time-frequency property of ECG beat, our work aimed to conduct tentative research on low-dimensional wavelet feature extraction and classification performance with the training set mixed with or independent from the test set. For the beat-based training scheme, our method could achieve better classification results than those of most of the investigated literatures. While for the record-based training scheme, the SVM classifier failed to perform effectively with the proposed features, no matter the feature dimension was reduced or not.

Our algorithm is compared with the related works over recent years, as illustrated in Table 6. All works compared here use the MITDB ECG data. It should be noted that these works use different number of classes, records, amount of data, and signal conditioning methods. All these factors can affect the classification performance. Besides, few publications evaluate their methods with the test set separated from the training set. Classifiers in most of the listed literatures are trained by methods similar to the beat-based scheme. As can be seen from Table 6, the most widely used feature extraction methods and classifiers are wavelet transform and SVM. Although many literatures have achieved high recognition rates, e.g. greater than 90%, with different feature dimensions, most of them test their algorithms over small datasets. Different from them, our method utilizes all the effective beats from MITDB and has achieved high classification performance with fewer features. The comparison demonstrates that the proposed scheme has the potential for solving the problem of ECG beat recognition and can be considered as a powerful tool for automatic cardiac arrhythmia classification.

**Table 6:** Comparison between the related works and the method proposed in this study.

Literatures and feature extraction methods	Feature selection (dimension)	Beat types	Training/test beats	Classifiers	Independent training/test data	k-fold cross validation	*SEN* (%)	*SPE* (%)	*ACC* (%)
Spectral correlation^1^	Yes (88)	5	Totally 6259	SVM	Unknown	10-fold	99.20	99.70	98.60
Wavelet transform, morphological features^5^	No (28)	5	10675/93894	Artificial neural network	No	No	88.60	96.18	97.86
Morphological features^7^	Yes (6)	6	35848/35848	Linear discriminant analysis	No	No	91.19	98.65	94.03
Morphological features^8^	No (13)	3	600/30273	SVM, neural network	No	No	98.52	99.19	97.14
Time domain features^9^	No (9)	6	42427/14142	Decision tree	No	No	97.50	99.80	99.51
Morphological features^10^	No (16)	3	15509/8081	SVM, neural network	Yes	No	92.82	93.74	92.85
Morphological features^11^	No (8)	5	12570/12570	Regression neural network	No	No	85.50	99.40	99.40
Fourier transform, wavelet package^14^	Yes (70)	16	3345/2542	*k*-NN	No	No	85.59	99.56	93.59
Wavelet transform, cosine transform^15^	Yes (18)	4	720/360	SVM	Unknown	No	98.60	95.50	96.50
Wavelet transform^16^	Yes (24)	5	900/900	SVM, genetic algorithm	No	No	98.50	99.69	98.80
Higher order spectral^17^	No (7)	5	330/500	SVM	Unknown	No	90.00	87.93	85.79
Wavelet transform^18^	Yes (20)	4	360/360	SVM	Unknown	No	98.62	99.54	98.61
Temporal and spectral features^21^	Yes (15)	6	1440/720	SVM	No	No	97.60	93.80	95.20
Temporal and spectral features^22^	Yes (13)	8	Totally 17857	SVM	No	5-fold	95.00	99.00	98.60
Higher order statistics, wavelet packet^27^	Yes (28)	5	3345/2542	*k*-NN	Yes	No	89.80	97.80	—
Hilbert-Huang transform^32^	Yes (18)	6	10700/10700	SVM	No	No	98.64	99.77	99.51
Wavelet transform^46^	Yes (18)	5	Totally 101352	SVM	Yes	44-fold	—	—	86.40
		16	24100/86009		No	No	99.32	—	99.01
Approximate entropy, wavelet packet^47^	Yes (9)	5	145/145	SVM, PNN	Unknown	No	98.70	99.70	98.60
Non-linear and center-clipping transform^48^	No (5)	5	13640/13640	Wavelet neural network	No	No	98.78	99.70	98.78
Eigenvector method^49^	Yes (12)	4	360/360	Recurrent neural network	Unknown	No	98.89	99.25	98.06
Higher order statistics^50^	No (24)	5	4000/14299	RBF neural network	No	No	92.93	98.52	95.18
Geometrical features^51^	No (18)	7	4035/3150	SVM, *k*-NN, BPNN	No	No	97.52	99.65	98.06
Wavelet transform, morphological features^52^	Yes (8)	3	50928/49636	Linear discriminant analysis	Yes	No	80.00	—	94.00
Wavelet transform, linear prediction model^53^	No (12)	3	50554/49273	Linear discriminant analysis	Unknown	No	86.50	—	86.50
Cross correlation^54^	No (30)	3	41961/51285	Artificial neural network	Unknown	No	97.49	—	95.24
**WMRA [This work]**	**Yes (12)**	**6**	**Totally 107049**	**SVM**	**Yes**	**10-fold**	**44.40**	**88.88**	**81.47**
					**No**		**99.09**	**99.82**	**99.70**

After PCA procedure, the feature dimension has decreased to 3.2% of the original dimension, while SVM property is not affected at all and achieves high classification rates for the beat-based training scheme. The substantial reduction of feature dimension indicates two critical issues. On one hand, wavelet coefficient from WMRA contains considerable redundancy, although it is regarded as one of the best feature extraction methods that can comprehensively represent time-frequency characteristics of an ECG signal. The redundant features may provide repetitive or even contradictory information, which may give rise to misleading the construction of a precise classification model. On the other, the classification process would be more efficient in training and prediction procedure with less but exhaustive features, without any degeneration in classification property.

With respect to the record-based training scheme, an effective solution for the imbalanced beats is to test different penalties for the margin slack variables of each class via minimizing the incorrectly classified beats by cross validation. But rigorous penalty factor results in over-fitting classification, because SVM training will construct extremely accurate hyper-plane that can separate all training beat samples thoroughly without any misclassification.

Another reason for the ineffective recognition of record-based training scheme is the irrelevant beats from unknown test set. In beat-based training scheme, chances are that beats in training and test set may come from the same ECG record. Although the training beats are also imbalanced, they have intensive relevance with test beats which can be accurately recognized by SVM. While in record-based training scheme, test set is completely independent from the training set with no interrelation. If each training beat type does not contain diverse waveforms, it would be difficult for the SVM to identify an unknown beat due to lack of comprehensive knowledge over the training beats. The following points are potential approaches to improve the recognition performance of classifiers: 1) Collecting more beats, especially for the unusual beat types; 2) Improving or developing multi-class classification methods for imbalanced learning that will consider varying relationships between classes, such as deep learning; 3) Focusing on the structure and nature of ECG beats in minority classes to gain a better insight into the source of learning difficulties; 4) Introducing efficient clustering methods for unevenly distributed beat groups and measures to properly evaluate and select partitioning models in such scenarios.

The differences between the numerous published works and this research are as follows.

1. Firstly, features were not extracted from wavelet coefficients directly in our study. Instead, they were obtained by transforming the wavelet coefficients via PCA. This is different from the traditional wavelet transform-based method that uses feature selection to reduce the feature dimension. In our work, PCA was directly performed on the wavelet coefficients for dimensionality reduction, therefore our work did not include a feature selection step, as shown in Fig. 7, where the left-hand side showed the traditional scheme while the right-hand side showed the scheme used in our work.

**Figure 7:**
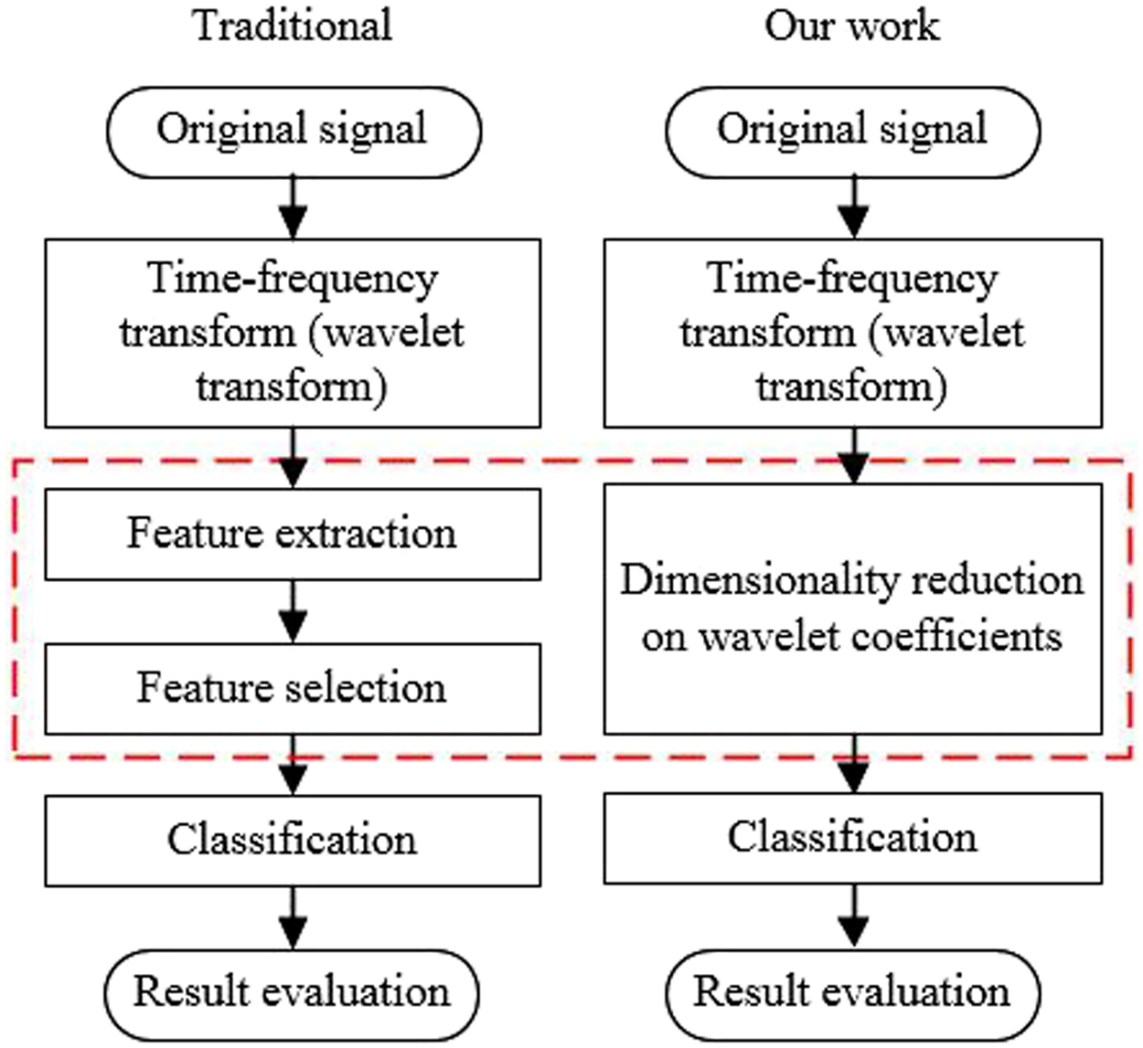
Comparison between our method and traditional wavelet transform-based methods.
2. Secondly, most of the literatures only considered wavelet coefficients in some certain levels, while in our study, all detail coefficients from 1st to 8th level were used. Besides, in many literatures, low-dimensional features were separately collected from each of the independent sub-frequency bands, while in our study, all the detail coefficients were arranged into a 1-D temporary vector, and PCA was directly applied to this temporary vector for dimensionality reduction by choosing the first few principal components with high contributory ratios.

3) Most importantly, the beat-based training scheme may result in over-fitting problem since the training and test beats may be contributed by the same patient. In our study, apart from the beat-based training scheme, a record-based scheme was also investigated, i.e., the classifier was trained and tested on totally separate records from different individuals. This is the most important aspect of our proposed study since other researchers usually verify the high performance of classifiers using the beat-based scheme without the verification of the record-based scheme.

As a most recently developed method, deep learning is attracting more and more attention due to its self-optimization over input features. This property may improve the classification rates of record-based training scheme, since deep learning inherently fuses “feature extraction” and “classification” as an integrated one and directly constructs a decision-making function. Although researchers have exerted plenty of effort to develop high-performance classifier, enriching samples of each arrhythmia type is still the most effective and fundamental approach that is almost always overlooked. Developing new classification structure is an effective measure to improve classification accuracy, however, the performance of a training model still depends significantly on the training data.

## Conclusions

This study describes a method of automatic feature extraction and classification for ECG beat. In feature extraction, ECG signals are segmented into 0.7 s beat episodes based on the R-peak locations. Wavelet coefficients are then collected by implementing WMRA on the episodes. The dimension of the full coefficients is reduced by PCA to obtain low-dimensional but efficient feature vectors. In classification, one-versus-one SVM combined with 10-fold cross validation is employed to recognize six types of ECG beats using a set of 12-element feature vectors. Tested upon a total of 107049 ECG beats, our work obtains a promising classification performance for the beat-based training scheme, but less effective performance for the record-based training scheme. The influence of the two training schemes on SVM classifier is also discussed. Compared with other techniques, our method is proven to be an effective alternative for automatic ECG feature extraction and classification to promote daily ECG monitoring. In future developments, we plan to increase the classification accuracy over the record-based training scheme in a number of ways, for example: (i) combining feature selection and feature dimensionality reduction processes; (ii) using the state-of-the-art deep learning method for classification of more types of heartbeats.

## Electronic supplementary material

Supplementary Information
