# Supplementary material for: Combining Low-dimensional Wavelet Features and Support Vector Machine for Arrhythmia Beat Classification
Source: Sci Rep. 2017 Jul 20;7:6067. doi: 10.1038/s41598-017-06596-z (PMC5519637; doi:10.1038/s41598-017-06596-z)
Supplement: Supplementary file 1 — Supplementary Information [file 41598_2017_6596_MOESM1_ESM.pdf]

Supplementary Information

Combining Low-dimensional Wavelet Features and Support Vector Machine for Arrhythmia Beat Classification

Qin Qin[1], Jianqing Li[1*], Li Zhang[2], Yinggao Yue[1], Chengyu Liu[1]

[1]School of Instrument Science and Engineering, Southeast University, Nanjing, 210018, P. R. China

[2]Computational Intelligence Research Group, Department of Computing Science and Digital Technologies, Faculty of Engineering and Environment, University of Northumbria, Newcastle, NE1 8ST, UK

*Corresponding author: ljq@seu.edu.cn (Jianqing Li), Tel: +8613809030922, Fax: +86-25-86508960

Email address: qq_nj@seu.edu.cn (Qin Qin), li.zhang@northumbria.ac.uk (Li Zhang), yyg@seu.edu.cn (Yinggao Yue), bestlcy@sdu.edu.cn (Chengyu Liu)

Supplementary Table S1. Classification results for the beat-based cross validation

1st	A	L	N	P	R	V	Beats	2nd	A	L	N	P	R	V	Beats
A	215	0	37	0	1	1	254	A	211	0	38	0	4	1	254
L	0	804	3	0	0	0	807	L	0	803	2	0	0	2	807
N	4	2	7476	0	0	20	7502	N	7	4	7469	0	1	21	7502
P	0	0	0	701	0	1	702	P	0	0	1	701	0	0	702
R	1	0	5	0	719	0	725	R	2	0	6	0	716	1	725
V	1	3	9	0	0	699	712	V	1	0	12	0	0	700	713
**3rd**	A	L	N	P	R	V	Beats	**4th**	A	L	N	P	R	V	Beats
A	217	0	33	0	3	1	254	A	211	0	36	0	6	1	254
L	0	803	1	0	0	3	807	L	0	799	4	0	0	4	807
N	11	0	7477	0	0	14	7502	N	5	0	7477	0	2	18	7502
P	0	0	0	702	0	0	702	P	0	0	0	702	0	0	702
R	2	0	1	0	722	0	725	R	1	0	1	0	720	3	725
V	1	1	15	0	0	696	713	V	0	4	17	1	1	690	713
**5th**	A	L	N	P	R	V	Beats	**6th**	A	L	N	P	R	V	Beats
A	206	1	46	0	0	2	255	A	212	0	39	0	4	0	255
L	0	803	2	0	0	2	807	L	0	799	2	0	0	6	807
N	5	4	7478	0	1	14	7502	N	15	0	7473	0	3	11	7502
P	0	0	0	701	0	1	702	P	0	0	1	702	0	0	703
R	0	0	0	0	722	3	725	R	2	0	2	0	722	0	726
V	1	1	12	1	1	697	713	V	1	0	13	0	0	699	713
**7th**	A	L	N	P	R	V	Beats	**8th**	A	L	N	P	R	V	Beats
A	221	0	32	0	1	1	255	A	206	0	46	0	3	0	255
L	1	801	1	0	1	3	807	L	0	800	5	0	0	2	807
N	7	1	7478	0	0	16	7502	N	3	1	7479	0	0	19	7502
P	0	0	0	701	0	2	703	P	0	0	0	701	0	2	703
R	1	0	4	0	721	0	726	R	2	0	5	0	718	1	726
V	0	4	22	1	0	686	713	V	1	0	22	1	0	689	713
**9th**	A	L	N	P	R	V	Beats	**10th**	A	L	N	P	R	V	Beats
A	211	0	41	0	1	2	255	A	212	0	40	0	2	1	255
L	0	803	4	0	0	1	808	L	0	802	4	0	0	2	808
N	5	0	7491	0	1	6	7503	N	8	1	7476	0	1	17	7503
P	0	0	0	703	0	0	703	P	0	0	0	702	0	1	703
R	3	0	3	0	718	2	726	R	1	0	1	0	724	0	726
V	1	3	11	0	0	698	713	V	2	1	16	1	0	693	713

Supplementary Table S2. Classification rates for the beat-based cross validation

**1st**	*SEN* (%)	*SPE* (%)	*ACC* (%)	**2nd**	*SEN* (%)	*SPE* (%)	*ACC* (%)
A	84.65	99.94	99.58	A	83.07	99.90	99.50
L	99.63	99.95	99.93	L	99.50	99.96	99.93
N	99.65	98.31	99.25	N	99.56	98.16	99.14
P	99.86	100.00	99.99	P	99.86	100.00	99.99
R	99.17	99.99	99.93	R	98.76	99.95	99.87
V	98.17	99.78	99.67	V	98.18	99.75	99.64
**Average**	99.18	99.84	99.73	**Average**	99.04	99.81	99.68
**3rd**	*SEN*	*SPE*	*ACC*	**4th**	*SEN*	*SPE*	*ACC*
A	85.43	99.87	99.52	A	83.07	99.94	99.54
L	99.50	99.99	99.95	L	99.01	99.96	99.89
N	99.67	98.44	99.30	N	99.67	98.19	99.22
P	100.00	100.00	100.00	P	100.00	99.99	99.99
R	99.59	99.97	99.94	R	99.31	99.91	99.87
V	97.62	99.82	99.67	V	96.77	99.74	99.54
**Average**	99.20	99.84	99.73	**Average**	99.03	99.81	99.68
**5th**	*SEN*	*SPE*	*ACC*	**6th**	*SEN*	*SPE*	*ACC*
A	80.78	99.94	99.49	A	83.14	99.83	99.43
L	99.50	99.94	99.91	L	99.01	100.00	99.93
N	99.68	98.13	99.22	N	99.61	98.22	99.20
P	99.86	99.99	99.98	P	99.86	100.00	99.99
R	99.59	99.98	99.95	R	99.45	99.93	99.90
V	97.76	99.78	99.64	V	98.04	99.83	99.71
**Average**	99.09	99.82	99.70	**Average**	99.08	99.82	99.69
**7th**	*SEN*	*SPE*	*ACC*	**8th**	*SEN*	*SPE*	*ACC*
A	86.67	99.91	99.60	A	80.78	99.94	99.49
L	99.26	99.95	99.90	L	99.13	99.99	99.93
N	99.68	98.16	99.22	N	99.69	97.57	99.06
P	99.72	99.99	99.97	P	99.72	99.99	99.97
R	99.31	99.98	99.93	R	98.90	99.97	99.90
V	96.21	99.78	99.54	V	96.63	99.76	99.55
**Average**	99.08	99.82	99.69	**Average**	98.94	99.79	99.65
**9th**	*SEN*	*SPE*	*ACC*	**10th**	*SEN*	*SPE*	*ACC*
A	82.75	99.91	99.51	A	83.14	99.89	99.50
L	99.38	99.97	99.93	L	99.26	99.98	99.93
N	99.84	98.16	99.34	N	99.64	98.10	99.18
P	100.00	100.00	100.00	P	99.86	99.99	99.98
R	98.90	99.98	99.91	R	99.72	99.97	99.95
V	97.90	99.89	99.76	V	97.19	99.79	99.62
**Average**	99.22	99.84	99.74	**Average**	99.08	99.82	99.69

Supplementary Table S3. Classification results for the record-based cross validation

**1st**	A	L	N	P	R	V	Beats	**2nd**	A	L	N	P	R	V	Beats
A	2	0	35	0	0	95	132	A	0	0	2	0	0	0	2
L	0	0	1038	0	0	1453	2491	L	409	0	1664	1	0	49	2123
N	2	0	4338	0	0	0	4340	N	7	0	3739	0	4	283	4033
P	0	0	4	0	0	1538	1542	P	0	0	5	0	0	2073	2078
R	0	0	144	0	0	2021	2165	R	2	1	1525	0	0	2	1530
V	0	1	38	0	0	178	217	V	5	8	112	0	0	543	668
**3rd**	A	L	N	P	R	V	Beats	**4th**	A	L	N	P	R	V	Beats
A	0	0	12	0	0	107	119	A	0	1	1	0	0	0	2
L	0	0	818	0	0	639	1457	L	520	0	969	0	1	511	2001
N	409	8	5882	0	8	0	6307	N	98	126	3292	0	3	1336	4855
P	0	0	1369	3	0	6	1378	P	1427	0	14	520	0	66	2027
R	0	0	85	0	0	0	85	R	0	0	1792	0	0	33	1825
V	0	0	65	1	0	84	150	V	0	0	52	13	0	304	369
**5th**	A	L	N	P	R	V	Beats	**6th**	A	L	N	P	R	V	Beats
A	0	0	1	0	0	0	1	A	1	1	1336	0	0	80	1418
L	0	0	993	0	0	1498	2491	L	1	0	906	10	0	1206	2123
N	246	0	3989	0	0	68	4303	N	22	17	3486	11	3	1294	4833
P	0	0	1490	0	0	588	2078	P	0	0	7	0	0	1535	1542
R	554	0	161	0	519	19	1253	R	0	0	390	0	1	6	397
V	5	1	18	0	0	78	102	V	0	5	128	0	2	491	626
**7th**	A	L	N	P	R	V	Beats	**8th**	A	L	N	P	R	V	Beats
A	13	0	371	0	0	202	586	A	0	0	12	0	0	0	12
L	1	0	628	0	0	828	1457	L	300	0	1209	0	0	492	2001
N	10	0	5118	0	9	3	5140	N	1	0	5432	0	0	6	5439
P	845	6	31	1049	0	96	2027	P	0	0	1369	3	0	6	1378
R	0	0	111	0	0	2139	2250	R	0	0	1529	0	0	1	1530
V	5	13	103	0	0	199	320	V	2	0	169	13	6	343	533
**9th**	A	L	N	P	R	V	Beats	**10th**	A	L	N	P	R	V	Beats
A	12	0	195	0	0	108	315	A	0	0	4	0	0	3	7
L	0	0	2017	0	0	1931	3948	L	241	0	1663	0	0	219	2123
N	73	0	2216	0	0	15	2304	N	0	147	3045	0	1	57	3250
P	0	0	6	0	0	1536	1542	P	1148	133	10	652	0	84	2027
R	0	0	85	0	0	0	85	R	0	0	1790	0	0	35	1825
V	0	0	93	1	3	208	305	V	0	1	67	0	0	767	835

Supplementary Table S4. Classification rates for the record-based cross validation

**1st**	*SEN* (%)	*SPE* (%)	*ACC* (%)	**2nd**	*SEN* (%)	*SPE* (%)	*ACC* (%)
A	1.52	99.98	98.79	A	0.00	95.95	95.93
L	0.00	99.99	77.11	L	0.00	99.89	79.57
N	99.95	80.77	88.42	N	92.71	48.32	65.48
P	0.00	100.00	85.84	P	0.00	99.99	80.07
R	0.00	100.00	80.11	R	0.00	99.96	85.30
V	82.03	52.14	52.73	V	81.29	75.35	75.73
**Average**	41.50	88.30	80.50	**Average**	41.04	88.21	80.35
**3rd**	*SEN*	*SPE*	*ACC*	**4th**	*SEN*	*SPE*	*ACC*
A	0.00	95.64	94.44	A	0.00	81.54	81.52
L	0.00	99.90	84.57	L	0.00	98.60	80.79
N	93.26	26.34	70.79	N	67.81	54.56	60.37
P	0.22	99.99	85.51	P	25.65	99.86	86.28
R	0.00	99.91	99.02	R	0.00	99.96	83.49
V	56.00	91.95	91.39	V	82.38	81.83	81.85
**Average**	62.86	92.57	87.62	**Average**	37.15	87.43	79.05
**5th**	*SEN*	*SPE*	*ACC*	**6th**	*SEN*	*SPE*	*ACC*
A	0.00	92.13	92.12	A	83.14	99.83	99.43
L	0.00	99.99	75.64	L	0.07	99.76	86.84
N	92.70	55.05	70.89	N	0.00	99.74	80.38
P	0.00	100.00	79.68	P	72.13	54.68	62.39
R	41.42	100.00	92.82	R	0.00	99.78	85.71
V	76.47	78.54	78.52	V	0.25	99.95	96.33
**Average**	44.84	88.97	81.61	**Average**	36.37	87.27	78.79
**7th**	*SEN*	*SPE*	*ACC*	**8th**	*SEN*	*SPE*	*ACC*
A	2.22	92.31	87.83	A	0.00	97.22	97.11
L	0.00	99.82	87.47	L	0.00	100.00	81.63
N	99.57	81.27	89.25	N	99.87	21.38	60.57
P	51.75	100.00	91.70	P	0.22	99.86	87.26
R	0.00	99.91	80.82	R	0.00	99.94	85.90
V	62.19	71.48	71.23	V	64.35	95.13	93.62
**Average**	54.15	90.83	84.72	**Average**	53.04	90.61	84.35
**9th**	*SEN*	*SPE*	*ACC*	**10th**	*SEN*	*SPE*	*ACC*
A	3.81	99.11	95.58	A	0.00	86.19	86.13
L	0.00	100.00	53.55	L	0.00	96.46	76.12
N	96.18	61.32	70.77	N	93.69	48.16	62.86
P	0.00	99.99	81.84	P	32.17	100.00	86.34
R	0.00	99.96	98.96	R	0.00	99.99	81.86
V	68.20	56.19	56.62	V	91.86	95.69	95.37
**Average**	28.66	85.73	76.22	**Average**	44.34	88.87	81.45

Appendix

The wavelet used in this study is *bior*6.8 wavelet, which is one of the biorthogonal and symmetric wavelets. It matches the intrinsic structure of heartbeat waveform and can achieve better denosing performance than other wavelets [13]. The property of symmetry ensures that it has linear phase characteristics. In fact, there are several kinds of wavelet functions that can be used for WMRA. The morphologies of frequently used wavelet functions are shown in Fig. A1. The red solid line and the blue dash line in each sub-figure denote the wavelet function and the scaling function, respectively, of each wavelet.

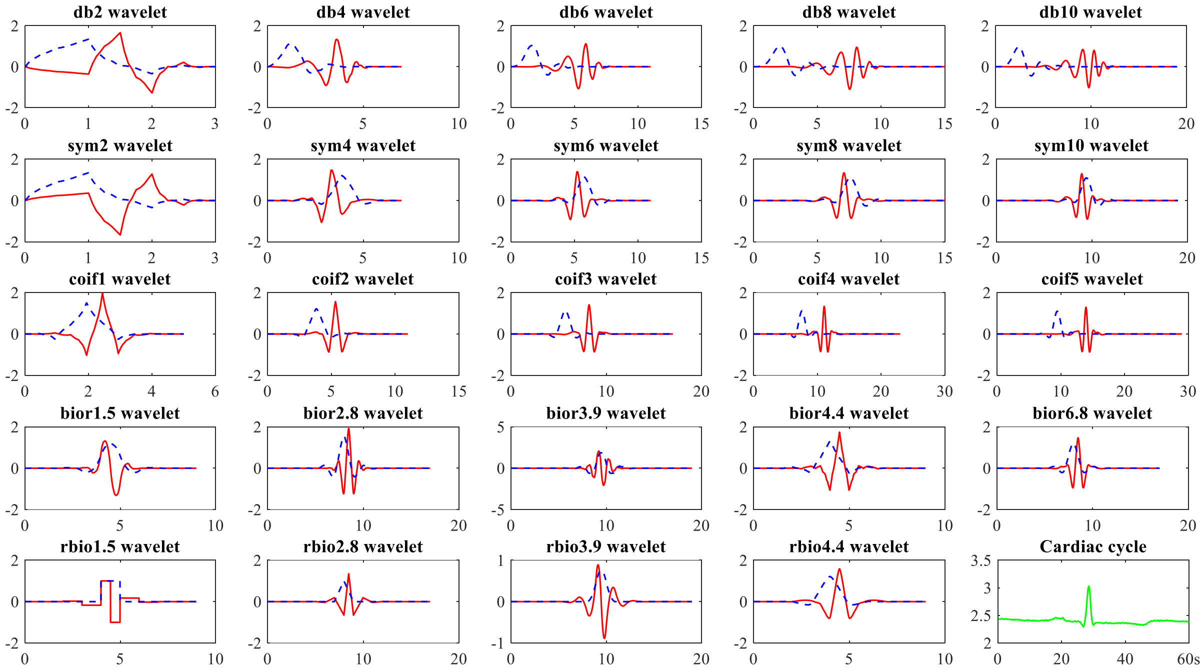

Fig. A1. Delineation of frequently used wavelets and one cardiac beat.

To reveal the property of *bior*6.8 wavelet, the shrinking threshold algorithm of WMRA described in [30] is implemented in signal denoising for Record 106 from MITDB. This record contains different types of interference such as baseline drift, muscle noise and motion artifacts. The denoising results are evaluated by signal-to-noise ratio (SNR), as defined in Eq. (A1) [30].

$$SNR = 10\,log_{10}\frac{\sum_{i=1}^{n} d^2(i)}{\sum_{i=1}^{n}[x(i)\text{-}d(i)]^2} \tag{A1}$$

where $x(n)$ is the original signal; $d(n)$ is the denoised signal; n is the signal length. Table A1 summarizes the SNR of Record 106 processed by different wavelet functions. It is obvious that *bior6.8* wavelet has realized the highest SNR among all the wavelets. Thus, in this study, *bior6.8* wavelet is adopted for WMRA enhancement.

Table A1. Denoising performance of frequently-used wavelets.

wavelet	SNR	wavelet	SNR	wavelet	SNR	wavelet	SNR
db1	18.257	sym1	18.257	bior1.1	18.257	rbio1.1	18.257
db2	15.938	sym2	15.938	bior1.3	17.788	rbio1.3	18.472
db3	15.414	sym3	15.414	bior1.5	17.637	rbio1.5	18.492

db4	16.235	sym4	26.489	bior2.2	26.515	rbio2.2	24.972
db5	18.337	sym5	16.015	bior2.4	27.178	rbio2.4	26.360
db6	21.776	sym6	28.131	bior2.6	27.325	rbio2.6	26.809
db7	24.433	sym7	16.671	bior2.8	27.279	rbio2.8	26.936
db8	21.695	sym8	28.323	bior3.1	14.260	rbio3.1	16.336
db9	18.627	sym9	16.145	bior3.3	15.800	rbio3.3	17.593
db10	16.899	sym10	27.871	bior3.5	15.964	rbio3.5	17.876
db11	16.244	sym11	18.188	bior3.7	15.988	rbio3.7	18.046
db12	16.407	sym12	15.412	bior3.9	15.995	rbio3.9	18.121
db13	17.250	sym13	18.068	bior4.4	28.332	rbio4.4	27.243
db14	18.632	sym14	15.404	bior5.5	16.317	rbio5.5	14.152
db15	20.127	sym15	17.613	***bior6.8***	***28.775***	rbio6.8	28.112
db16	20.896	sym16	15.422	coif1	27.209		
db17	20.239	sym17	17.667	coif2	27.758		
db18	18.794	sym18	27.557	coif3	26.946		
db19	17.543	sym19	16.928	coif4	25.686		
db20	16.923	sym20	15.461	coif5	24.562		
